# Passive smoking, as measured by hair nicotine, and severity of acute lower respiratory illnesses among children

**DOI:** 10.1186/1617-9625-1-4

**Published:** 2002-01-15

**Authors:** WK Al-Delaimy, J Crane, A Woodward

**Affiliations:** 1 Department of Public health, Wellington School of Medicine, Wellington, New Zealand; 2 Department of Medicine, Wellington School of Medicine, Wellington, New Zealand; 3 Department of Nutrition, Harvard School of Public Health, Boston, MA 02115, USA

## Abstract

The aim of this study was to describe the association between passive smoking and the severity of acute lower respiratory illnesses (ALRI) among 351 children aged 3–27 months admitted to hospital. A total of 297 children provided hair samples, which were analysed for hair nicotine levels as an indicator of passive smoking. A severity of illness grading system was developed by using clinical and management criteria used by the medical staff at hospital. The OR for children with more severe illness being exposed to higher nicotine levels was 1.2, 95% CI: 0.57–2.58 when using dichotomised respiratory severity levels and upper versus lower nicotine quartile levels. In an ordinal logistic regression model, the OR of more severe illness being associated with higher nicotine levels was 1.07 (95% CI: 0.92–1.25). When analysis was limited to the more severe cases, the OR of the least severe category compared to the most severe category, in relation to nicotine levels in hair, was 1.79 (95% CI: 0.5–6.30). The ordinal logistic regression of this group of severely-ill children (OR 1.1 (95% CI: 0.94–1.29) was not substantially different from the overall study subjects.

In general, children with more severe illness tended to have higher levels of nicotine in their hair, although the results were within the limit of chance. Possible explanations of our results include environmental tobacco smoke (ETS) being an initiator of ALRI rather than a risk to severity, exposure levels of ETS were too low to demonstrate an effect on severity, or the power of this study was not high enough to detect an association.

## Introduction

Although many studies have examined the relationship between passive smoking and the incidence of lower respiratory illnesses [1,2], few have looked at the severity of these illnesses in young children in relation to passive smoking [3-5] and a lesser number of studies [6] used an objective continuous measure of environmental tobacco smoke (ETS) exposure.

Difficulties in ETS exposure measurement have been a considerable obstacle for precise interpretation of the associations between ETS and illnesses. Questionnaires usually do not provide high sensitivity and objectivity in estimating exposure to ETS due to recall and reporting bias [7]. Furthermore, the available biomarkers only measure short-term exposure (hours to few days), which makes them vulnerable to error produced by the fluctuating nature of ETS exposure. However, a recent biomarker, hair nicotine, has been shown to overcome these problems. Because of the slow hair growth rate (approximately 1 cm/month), each 1 cm of hair is an estimation of one month of previous exposure to ETS [8]. Therefore it provides a relatively long-term measure, which is less vulnerable to day-to-day variation of ETS exposure and can give a more accurate estimate of the average exposure of an individual [9-12]. In addition, it is easily collected and stored.

The aim in this study was to include children admitted to hospital with relatively severe ALRI (acute lower respiratory illnesses) and measure their exposure to ETS by analysing their hair samples in an attempt to assess the association between ETS exposure and severity of ALRI.

## Patients and Methods

The reference populations of this cross-sectional study were all children consecutively admitted to one of the three general hospitals in Wellington district (Wellington, Hutt Valley, and Kenepuru) during the period of the study. Children admitted with ALRI to these Wellington regional hospitals were eligible to enter the study if they were aged two years or less at the time of admission. This age group was chosen since earlier studies in New Zealand and elsewhere have indicated that the relationship between ETS and ALRI is strongest in children under two years of age [13,14]. The three hospitals are the only regional children's hospitals serving the area of the lower North Island and upper South Island of New Zealand. Infants under 3 months were excluded to avoid lower respiratory diseases secondary to neonatal problems [15].

Parents and caregivers of 351 children agreed to take part in the study that was carried out from August 1997 until October 1998. Fifty four children provided questionnaire data but no hair samples, and were therefore excluded. Thirteen children who were involved in the study and provided hair samples were 1–3 months above the intended upper age limit of 24 months. Those were not excluded from the study analysis.

Children who had a preliminary diagnosis of bronchiolitis, pneumonia, asthma, or croup made by the admitting paediatrician were included in the study. These illnesses represent ALRI according to the WHO criteria [16]. The final diagnosis was modified in 17 (5.3%) children to upper respiratory illness; those children were therefore excluded from the initial statistical analysis of the association between ETS and ALRI, but were re-included in the secondary analysis for assessment of children according to their severity signs (discussed later in the results). Children with known congenital abnormalities or cystic fibrosis were not enrolled.

### Study protocol

Each morning during the period of the study, newly admitted children were screened by one of the study investigators for inclusion in the study. Children whose clinical notes suggested a chief complaint of cough, rapid or difficult breathing, wheezing, or signs of chest rales/crepitations [17,18], were considered eligible for the study. Children with a diagnosis made by the admitting physician of pneumonia (diagnosed by chest x-ray), asthma (previous diagnosis of asthma, on asthma medication, or expiratory wheeze), croup (inspiratory stridor) or bronchiolitis (crackles at lung bases) were also included.

Parents of the children were approached and their consent to participate in the study was sought. A 5–10 minutes interview with the parent was then carried out to record information about the history of the illness. In addition, household smoking history was recorded to validate the hair nicotine method, which was found highly correlated to questionnaire smoking history [9]. Nicotine in hair was found to be better correlated with history of exposure than cotinine in hair [10]. Data were collected on possible confounders such as age, crowding (measured here by dividing the number of people in the house by the number of rooms in the house), breastfeeding history, smoking history during pregnancy, number of children in the house, type of cooker, type of heating, and respiratory distress or prematurity in the neonatal period, and other demographic information.

Afterwards, a hair sample was collected by cutting 10–50 mg of hair from the child's scalp as close as possible to the skin. The hair sample was held in one bunch and put in a coded small paper envelope with the cut end of the hair in first. Hair samples were stored up to a year before being analysed. Other investigators have found nicotine levels in hair samples do not undergo loss or degradation even after several years of storage in paper envelopes at normal room temperature [11]. Hair samples were blinded to the laboratory, and analyses of the samples was performed by High Performance Liquid Chromatography with Electrochemical detection (HPLC-ECD), which we developed and found to have a high sensitivity (<0.05 ng/mg) and relative standard deviation <10% [19]. Furthermore, among 78 children, we were able to collect a second hair sample from them after 3–6 months to assess the biological variability and reliability of the biomarker.

The research staff then reviewed the medical records of the patient for the doctor and nurse's clinical notes on admission. The date of admission, respiratory rate, oxygen saturation, temperature, and heart rate on admission were collected from the nurse's admission notes. After discharge of the patient, the notes were again reviewed for the hospital admission duration, the need for oxygen supplementation, and final diagnosis by the paediatrician.

The NZ Dep classification (decile 1 is the highest socioeconomic status (SES) or least deprived while decile 10 is the lowest SES or most deprived), a measure of social and economic deprivation of small areas [20], was applied in the study based on the street addresses of each child. The Wellington Ethics Committee and groups representing local Maori approved the study protocol.

### Severity of the illness

There is challenge in assessing the severity of illnesses of children with ALRI. There is no gold standard used for such a purpose. However, chest indrawing, respiratory rate and the presence of danger signs have been used for the last two decades by the WHO as a simple clinical measure of the severity of ALRI [21,22]. O_2 _saturation and supplementation and duration of admission have also been shown to be good indicators of the severity of ALRI [23-26].

It was decided to combine all the signs and severity criteria in one clinical grading system to obtain a more robust measure of severity (see Table 1). This judgement was based on the experience of other researchers. For example, Leventhal [27] found that out of 19 clinical signs associated with pneumonia in children, no single sign predicted pneumonia when used individually, while a combination of signs was a strong diagnostic indicator of pneumonia.

**Table 1:** The severity categories according to the clinical signs and management criteria

Severity group	Respiratory rate (RR) [>50/min^*a *^or >40/min^*b*^]	Chest Indrawing	SaO_2 _%^*c*^	0_2 _supplement (hours)	Days of admission	ICU admission	CPAP
1	-^*d*^	-	>95	-	1	-	-
2	+^*e*^	Intercostal	>95	-	1–2	-	-
3	±^*f*^	subcostal	>95	-	2–3	-	-
4	±	±	91–95	<48	4–5	-	-
5	±	±	<91	≥ 48	>5	+	+

^*a*^: RR per minute for children under the age of 12 months.

^*b*^: RR per minute for children aged 12 months or more.

^*c*^: oxygen saturation percent in plasma.

^*d*^: a negative finding (absent)

^*e*^: a positive finding (present).

^*f*^: finding may be present or absent (i.e. becomes irrelevant for that specific severity group).

The first requirement when developing categories for a new measure is that they should make sense based on prior knowledge [28]. The categories used in this study were created following a review of the published literature. Although the same combination of criteria have not been used previously, the components have been used either separately or in combination in other scoring systems [29-31]. Severity, in this study, was regarded as an indication of the level of disability of the child with ALRI. Sixty eight children were clinically assessed by a paediatrician for their ALRI severity. This subgroup was used for comparison with the severity grading system developed in this study.

### Data Analysis

The Spearman rank correlation was calculated to assess the strength of the association between severity categories and paediatrician severity assessment, and for the association between two hair samples taken from the same children 3–6 months apart. This test is appropriate for comparing ordinal variables similar to the severity categories used in this study [32].

The Kruskall-Wallis rank test was used to assess the significance in the difference of nicotine levels among the severity categories, and the difference of severity (the five ordered categories considered numerical) among non-ordered categories of ethnicity and ALRI diagnosis as risk factors. The Wilcoxon rank test was used when the severity was dichotomised in relation to ETS exposure.

The Mantel trend statistic was used for assessment of the association of ALRI risk factors and severity of ALRI. Dichotomised categories of ALRI severity were examined in relation to ETS exposure by Chi-square and Cochrane-Mantel-Haenszel (CMH) tests, adjusting for other variables in the relationship. Continuous variables (nicotine, SES, crowding) were dichotomised at their median value (≤ 2.33, >2.33 ng/mg hair for nicotine; decile 7, > decile 7 for SES; and ≤ 1.5, >1.5 ratio value for crowding).

Ordinal logistic regression modelling [28] was also used for assessment of the relationship of ETS exposure with ALRI severity, controlling for possible confounders. This method is used when a dependent variable (ALRI severity) has more than 2 ordered categories. This was done using the "LOGISTIC" procedure in SAS [33], and sorting the severity categories so that the most severe category becomes the reference group. The odds ratio and 95% confidence limits were produced for each regressor in the model and the effect of confounding was assessed in relation to the overall model.

## Results

The ethnic distribution of children in the study sample was: 30.1% identified with being solely or partially of Maori ethnicity, 32% identified with being solely or partially of Pacific Island ethnicity (excluding Maori ethnicity), 31.4% identified as New Zealanders of European origin, 6.5% were all other ethnicities.

Bronchiolitis was the most common diagnosis of patients in this study (n = 149), followed by pneumonia (n = 66), asthma (n = 58) and croup (n = 37) respectively. Very few patients needed continuous positive airway pressure (CPAP), and only 3 patients were admitted to ICU. Chest indrawing was recorded for more than 70% of patients, however in only 56% was a specific category (intercostal, subcostal) of indrawing recorded.

Hair samples from 78 children taken 3–6 months apart and with no reported change on ETS exposure were strongly correlated (r = 0.92 p < .0001)

### Validity of severity categorisation

The face validity of the severity grading system is supported by earlier published research. In Table 2 it is evident that children who were diagnosed with pneumonia, bronchiolitis or asthma, were on the whole more severely affected, while most of the croup patients were categorised as least severe, as would be expected from the nature of these illnesses. The association between the severity categories and the severity of 68 children as assessed by the paediatrician (considered by some authors as the best available severity criteria [34-36]) was significantly and moderately correlated (r = 0.45, p < 0.0001).

**Table 2:** Distribution of the diagnosis of illness among the two categories of severity (1, 2 vs 3, 4, 5)

	Asthma	Bronchiollitis	Pneumonia	Croup
Severity categories 1 and 2 (less severe)	27.3%	30.2%	14.3%	62.2%
Severity categories 3, 4 and 5 (more severe)	72.7%	69.8%	85.7%	37.8%

### Severity categories and nicotine levels

**Figure 1:**
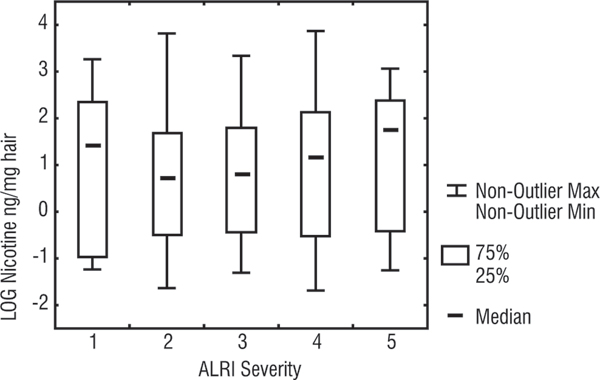
**Box Plot of nicotine hair levels according to ALRI severity categorisation**. Whiskers are the range of values (excluding outliers), Boxes are 25%–75% percentiles, dash in the box is the median, Y axis is the log of hair nicotine values for children, X axis is the 5 severity levels (level 1 is least severe and level 5 is the most severe).

Figure 1 and Table 3 show the distribution of nicotine levels according to the severity of ALRI. Except for the first (least severe) group, there is some indication of a positive trend with increasing exposure to ETS by severity of illness, but there is a great deal of overlap of hair nicotine levels between categories of severity.

**Table 3:** Median nicotine and cotinine levels for each respiratory severity category

Severity	N	Median	Nicotine Percentile (25%–75%)
1 (mild)	24	4.23	0.38–10.5
2	51	2.02	0.61–5.38
3	86	2.25	0.66–6.0
4	108	3.24	0.60–8.39
5 (severe)	12	6.01	0.70–10.99

Children with the least severe ALRI (group 1) did not differ from those in the other severity groups in the proportion of smoking mothers, number of smokers, total number of cigarettes, or the place of smoking at home (inside, outside). However they had a relatively high percentage (50%) of parents who smoked heavily (the sum of number of cigarettes smoked per day was more than 20 cigarettes per day), compared to most of the other categories: 32% of group 2, 47% of group 3, 40% of group 4, and 60% of group 5 had heavy smoking parents. In addition, 62.5% of the children in the least severe category lived in crowded houses (more than 1 person per room) while the other 4 groups had a range of 32 – 45%.

There were no statistically significant differences in the hair nicotine levels between the 5 severity categories (Kruskal Wallis; p = 0.81). Furthermore, restricting the distribution to children from houses where smokers lived did not change the trend. When the severity categories were dichotomised (categories 1 & 2 vs. categories 3, 4, and 5) there was a slight difference in the median hair nicotine levels (2.3, 2.4) but this may well have been a chance finding (Wilcoxon, p = 0.48). In a 2 × 2 table of the upper and lower quartiles of nicotine levels and the dichotomised severity levels of ALRI, the OR (for more severe illness given higher exposure to ETS) was 1.2 (CI: 0.57–2.58).

### Confounding

Only the age of the children and the diagnosis of the illness were associated with severity of ALRI among all possible confounders. History of breastfeeding was the only factor that when adjusted for, affected the OR (from 1.05 (CI: 0.62–1.78) to 1.15 (CI: 0.67–1.97)). When using the CMH test, severity and nicotine were dichotomised as described earlier. Severity of ALRI may vary according to season, being more severe in winter (when chest infections are most common) and early spring and less severe in summer. However, this was not found in this study (X^2 ^= 10.9, p = 0.54).

Using ordinal logistic regression modelling, the unadjusted OR of ETS being related to more severe ALRI was 1.07 (CI: 0.92–1.25). This model was further run controlling for possible confounders mentioned above, and differed little from the unadjusted OR.

### Analysis of children with positive severity signs only

The next step was to limit the analysis to children with positive physical signs or management criteria regardless of the physician diagnoses, in an attempt to focus on children who were presumably experiencing more severe illnesses.

Children excluded from this subgroup analysis were those with no chest indrawing, RR less than cut-off of severe ALRI, O_2 _saturation more than 95%, no supplemental O_2 _required, hospital stay for one-day only, and no ICU admission. In addition, the 17 children who were diagnosed by the paediatrician as having upper respiratory illness but had one of the severity signs were re-included in the analysis. Two hundred and sixty one children were considered eligible.

With this exclusion, the OR of the 2 × 2 table of severity and nicotine levels increased slightly (1.2 (CI: 0.7–1.8)), although the confidence interval still included the null value of one. Children in the least severe category were then compared to those in the most severe category, in relation to nicotine levels. The OR was 1.79 (CI: 0.5–6.3, n = 69) for more severe ALRI cases being exposed to higher nicotine levels.

The OR describing the strength of association between severity and nicotine levels in the ordinal logistic regression model increased very slightly (1.10 (CI: 0.94–1.29)) compared to earlier analysis of the total study population. The ALRI risk factors had similar relationships to severity as earlier, and there was no confounding effect from these variables on the association between severity and nicotine.

**Figure 2:**
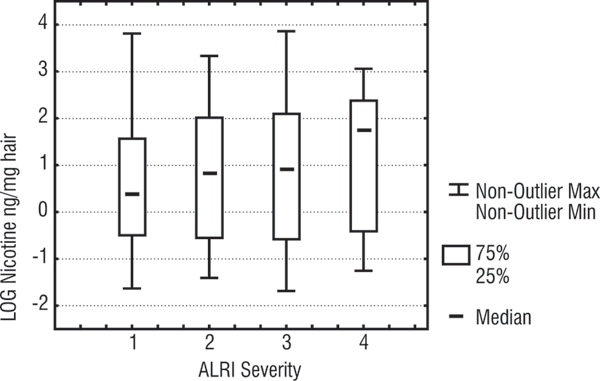
**Box plot of hair nicotine levels according ALRI severity (only children with positive severity signs included)**. Whiskers are the range of values (excluding outliers), boxes are 25%–75% percentiles, dash in the box is the median, Y axis is the log of hair nicotine values for children, X axis is the 4 severity levels (level 1 is least severe and level 4 is the most severe).

Although the hair nicotine levels were not significantly different across the four severity categories (Kruskal Wallis; p = 0.56), Figure 2 does show a monotonic increase according to the 4 severity categories. (The median nicotine levels were 1.5, 2.3, 2.6, and 6 ng/mg hair for severity groups 1, 2, 3, and 4 respectively.)

A retrospective power analysis in this group of children indicates that in order to have 90% power (probability) of detecting a true difference of 0.25 or more in log (nicotine levels) (1.28 ng nicotine/mg hair) between severity categories 1 & 2 and 3 & 4, and accepting a type I error of <0.05, at least 756 subjects would be needed in each comparison group.

## Discussion

Findings from this study show a weak trend of higher ETS exposure among children with more severe respiratory illnesses. However this did not reach statistical significance. Given the large number of factors that may influence severity of ALRI (virulence of the causative micro-organism, host immunity, type of medical intervention), it seems that the effect of ETS is overwhelmed by the effect of other factors and is difficult to detect using a study sample size similar to ours. The positive monotonic dose-response relationship observed in Figure 2 suggests there may be an underlying association between ETS and ALRI severity. A very much larger number of subjects would be needed to confirm that the association was real.

The least severe category of ALRI did not follow the pattern of the other four more severe categories in relation to ETS dose. In addition to having heavier smoking parents, possible explanations for the higher nicotine levels among the least severe group include their higher prevalence of first-time hospital admissions compared to the other groups (i.e. parents of first-time admitted children did not get advice on how to reduce exposure of their children). However, children admitted to hospital more than once had higher median nicotine levels (3.8 ng/mg hair) than those not previously admitted (2.3 ng/mg hair), which maybe explained by higher exposure of ETS being related to higher "incidence" of ALRI that require admission.

When children with no severity signs were excluded, the association between illness OR between severity and ETS exposure was strengthened somewhat. However, there was still a wide confidence interval around the risk estimates, including the null value of one. Since ETS appears to follow a positive trend with the more severe rather than the less severe cases of ALRI, it may be argued that studies that set out to detect a clinical difference in the severity of respiratory illnesses in relation to interventions or risk factors need to involve only the most severe cases [29].

### Possible explanations of the lack of a strong association

The study would have had greater statistical power if it included greater numbers at both ends of the severity spectrum. This would have improved the ability of the study to detect an association with ETS. To involve more of the less severe ALRI cases would require a study that included both hospital and community elements, since most of the least severe cases of ALRI probably do not reach hospital. The required sample size may not be feasible in a New Zealand setting, in which case a multi-centre study would be required. We chose a hospital setting rather than a community based study because hospital studies are likely to provide a more accurate diagnosis and more detailed clinical descriptions of the course of illness, because of the availability of specialist expertise and medical monitoring facilities [37]. Many measures of the severity of acute lower respiratory illnesses (ALRI) (such as O_2 _saturation, the requirement for supplementary oxygen) cannot be readily obtained outside hospital.

Another possible explanation for the finding of this study may be that constituents of ETS are initiators rather than aggravators of ALRI. Most studies in the literature reported an association between ETS and the incidence of ALRI. Incidence has been measured by the number of previous hospital admissions [38], parental reports [6,39], or paediatrician diagnosis and hospital records [40].

The effect of ETS on the respiratory system may be confined to the role of triggering an ALRI rather than sustaining and aggravating it. Among the several hypothesised mechanisms of effect of ETS on ALRI, Sun et al. [41] and Yates et al. [42] proposed that ETS irritates or damages the respiratory cell lining making it vulnerable to allergens or micro-organisms to penetrate the respiratory system and cause ALRI. The finding in this study of higher nicotine levels among children with previous hospital admission for respiratory illnesses compared to those who were admitted for the first time supports such an argument.

Another aspect to be considered is the relatively low levels of ETS exposure among children in this study. Wright et al. [40] for example, found that the OR of infants having an ALRI were approximately doubled if mothers smoked a pack of cigarettes or more per day. Most of the parents in this study reported that they smoked only 6 to 10 cigarettes per day. This level of smoking may not have been of sufficient magnitude to cause more severe ALRI cases, or show a dose-response relationship. Furthermore, it is theoretically postulated that the lack of a strong correlation between ETS and ALRI severity is due to large intra-individual variability of ETS exposure or unreliable assay. However, in our study we found the repeated tests of hair nicotine showed a close correlation between hair samples several months apart, and the assay has been validated in the laboratory and in the field [19], and the results cannot be explained by measurement error.

In conclusion, this study did not find a strong association between ETS and severity of ALRI, although there was a weak trend of a positive association. However, this was statistically non-significant. Possible explanations of our results include ETS being an initiator of ALRI rather than a risk to severity, exposure levels of ETS were too low to demonstrate an effect on severity, or the power of this study was not high enough to detect an association.

## Competing interests

The authors declare that they have no competing interests.
